# Endoscopic transorbital amygdalohippocampectomy for the resection of an epileptogenic lesion

**DOI:** 10.3171/2025.1.FOCVID24198

**Published:** 2025-04-01

**Authors:** Sami Obaid, Tristan Martin, Benjamin S. Kahozi, Guillaume Théaud, Béatrice Voizard, Pascal Lavergne

**Affiliations:** 1 Division of Neurosurgery, Department of Surgery, University of Montreal Hospital Center (CHUM), Montréal;; 2 Department of Surgery, University of Montreal;; 3 Faculty of Medicine, University of Montreal;; 4 University of Montreal Hospital Center Research Center (CRCHUM), Montréal;; Divisions of 5 Otolaryngology–Head and Neck Surgery, Montreal Sacred Heart Hospital, Montréal, Québec, Canada and; 6 Neurosurgery, Department of Surgery, Montreal Sacred Heart Hospital, Montréal, Québec, Canada

**Keywords:** endoscopic transorbital, amygdalohippocampectomy, minimally invasive surgery

## Abstract

Anterior temporal lobectomy and selective amygdalohippocampectomy are standard treatments for refractory temporal lobe epilepsy. Yet, they carry risks of lateral temporal damage to both neocortical and white matter structures, including the optic radiations. This video details a case of endoscopic transorbital amygdalohippocampectomy performed to resect an epileptogenic lesion. This approach offers minimally invasive access to the medial temporal lobe along its longitudinal axis, while preserving the lateral temporal area. Postoperative MRI showed resection of most of the amygdala, including its superior portion, and the hippocampus up to the tectal plate. This minimally invasive technique provides a promising alternative to traditional open surgery.

The video can be found here: https://stream.cadmore.media/r10.3171/2025.1.FOCVID24198

## Transcript

### 0:21 Patient Presentation.

This video presents an endoscopic transorbital amygdalohippocampectomy for the resection of an epileptogenic lesion. The patient is a 25-year-old male who presented with new-onset seizures of right mesial temporal origin. Seizure control was achieved with two antiseizure medications. Neuropsychological evaluation demonstrated well-compensated, slight visuospatial memory deficits. The MRI was consistent with a suspected infiltrative lesion of the right amygdala and hippocampus.

### 0:50 Classical Open Approaches.

Anterior temporal lobectomy and selective amygdalohippocampectomy are classic procedures commonly used to treat refractory temporal lobe epilepsy.^1^ While temporal lobectomy may offer superior seizure control,^2^ it is more invasive and requires resection of the temporal pole and basal temporal language area (BTLA), which may impact visual naming functions in the dominant hemisphere.^3^ There is also a risk of damage to the optic radiations, located superolaterally to the temporal horn, potentially leading to postoperative superior quadrantanopia.

Selective amygdalohippocampectomy will preserve most of the temporal neocortex. Yet, it is associated with retraction required to reach the posterior hippocampus as well as a risk of damage to the optic radiations.

### 1:36 Alternative Approaches.

Laser interstitial thermal therapy is a minimally invasive alternative to selective amygdalohippocampectomy allowing preservation of the lateral temporal neocortex via a minimally invasive stereotactic approach.^4^ However, it is associated with difficulty accessing the posterior part of the tail of the hippocampus, which curves around the tectal plate.

For these reasons, we proceeded with an endoscopic transorbital amygdalohippocampectomy. It allows preservation of the lateral temporal neocortex and optic radiations while following a surgical trajectory along the axis of the hippocampus. Furthermore, it provides better tissue sampling than LITT for tumors, it minimizes brain retraction, and it potentially achieves a more posterior hippocampal resection.

### 2:20 Endoscopic Transorbital Approach.

The procedure begins with an upper eyelid incision, followed by preseptal dissection^5^ toward the superior orbital rim. The periorbita is opened over the rim and dissected in a subperiosteal plane.^5^ The inner part of the superolateral portion of the rim is drilled to increase the maneuverability of the instruments. This is called a lacrimal keyhole.^6^

The subperiosteal dissection is then extended posteriorly toward the superior and inferior orbital fissures.^5^ The greater wing of the sphenoid is drilled to expose the temporal fascia laterally, and further drilling is performed between the superior and inferior orbital fissures to reveal the temporal pole dura posteriorly.^7^ Drilling continues to ensure sufficient exposure medially, and also toward the floor of the middle fossa, which is drilled extensively.

Once we are satisfied with our exposure, we confirmed our trajectory with the aid of neuronavigation. The dura is then coagulated and opened in a cruciate fashion to expose the temporal pole.

### 3:28 Amygdalohippocampectomy.

Once adequate exposure of the temporal pole is achieved, it is coagulated and an anterior temporal cortectomy is performed. The resection of the temporal pole is continued toward the temporal horn of the lateral ventricle with the aid of neuronavigation. Once the temporal horn has been found, it gives us a clear landmark to continue our dissection. Further opening of the temporal horn gave us a great view of the amygdala. We pursed our dissection and collected some specimens for pathologic analysis.

More resection of the temporal tip allowed us to continue our dissection posteriorly. We could then identify the hippocampus that was localized posterior to the amygdala. With the aid of the ultrasonic aspirator, we started the resection of amygdala. This resection allowed us to localize the body of the hippocampus as well as the choroid plexus. With these landmarks clearly identified, we continued our dissection medially, exposing the hippocampal sulcus. The subiculum and parahippocampal gyrus are then located inferiorly, and the hippocampus proper superiorly.^8^ The resection of the parahippocampal gyrus was pursued medially until we reached the basal cistern pia mater. The ultrasonic aspirator was used to continue the resection, and our position was confirmed with navigation.

The resection was also continued superiorly to the hippocampal sulcus where the hippocampus seemed infiltrated. Resection is also continued posteriorly and medially until we have identified the crus cerebri in a subpial fashion.

We then turned our attention laterally and opened the ventricle further, allowing identification of the collateral eminence, which served as the lateral border of our resection.^9^ Using this landmark, we proceed to resect the lateral hippocampus, including the posterior portion, which appeared less infiltrated. We identified the medial curve of the hippocampus and confirmed our location near the tectal plate using neuronavigation.

We then completed the medial and superior resection, further exposing the brainstem. We finished the resection by removing the most posterior part of the hippocampus and parahippocampal gyrus. We could then clearly view the choroid plexus and the inferior choroidal point, which were located more anteriorly. Upon final inspection, we completed the resection with the most medial part of the uncus that was remaining. After this final resection, we had a clear overview of the resection cavity and the basal cistern pia, which are well exposed.

### 6:42 Closure.

After achieving hemostasis, reconstruction is performed with a dual inlay and onlay synthetic graft, which is reinforced with an abdominal fat graft and fibrin glue.^10^ Closure is performed in the usual fashion with interrupted sutures on the periorbita and a running resorbable suture on the skin.

### 7:03 Postoperative Course.

The patient did well postoperatively with no new neurological deficits, except for transient diplopia.

The patient exhibited slight eyelid asymmetry, most likely secondary to subcutaneous scarring at 6 weeks postoperative. However, this does not result in ptosis, as the margin reflex distance is equal bilaterally.

The postoperative MRI revealed complete resection of the amygdala, the head and body of the hippocampus, and a substantial portion of the hippocampal tail up to the tectal plate, with complete preservation of the temporal stem.

The 2-month postoperative MRI with high-definition tractography revealed complete preservation of the optic radiations.

Clinically, the patient had no change in the neuropsychological evaluation. The visual fields were normal by confrontation, and formal visual fields are planned. He had no seizure recurrence, but the medication will be weaned at 6 months postoperative.

Pathology was negative for a glioma, but revealed some focal hypercellularity.

## Disclosures

The authors report no conflict of interest concerning the materials or methods used in this study or the findings specified in this publication.

## Author Contributions

Primary surgeon: Lavergne. Assistant surgeon: Obaid, Kahozi, Voizard. Editing and drafting the video and abstract: all authors. Critically revising the work: Lavergne, Obaid, Kahozi, Voizard. Reviewed submitted version of the work: Lavergne, Obaid, Voizard. Approved the final version of the work on behalf of all authors: Lavergne. Supervision: Lavergne, Obaid, Voizard.

## Supplemental Information

### Patient Informed Consent

The necessary patient informed consent was obtained in this study.
